# Development of an Efficiency Platform Based on MQTT for UAV Controlling and DoS Attack Detection

**DOI:** 10.3390/s22176567

**Published:** 2022-08-31

**Authors:** Leandro Marcos da Silva, Henrique Bonini de Britto Menezes, Matheus dos Santos Luccas, Christian Mailer, Alex Sandro Roschildt Pinto, Adão Boava, Mariana Rodrigues, Isadora Garcia Ferrão, Júlio Cézar Estrella, Kalinka Regina Lucas Jaquie Castelo Branco

**Affiliations:** 1 Institute of Mathematics and Computer Sciences, University of São Paulo, Ave. Trabalhador São-Carlense, 400, São Carlos 13564-002, São Paulo, Brazil; 2 Department of Informatics and Statistics, Federal University of Santa Catarina, St. Roberto Sampaio Gonzaga, Florianópolis 88040-900, Santa Catarina, Brazil

**Keywords:** unmanned aerial vehicles, Internet of Things, Message Queuing Telemetry Transport, artificial intelligence, machine learning, quality of service, security, denial of service

## Abstract

Several market sectors are attracted by the potential of unmanned aerial vehicles (UAVs), such as delivery, agriculture, and cinema, among others. UAVs are becoming part of Internet of Things (IoT) networks in the development of autonomous and scalable solutions. However, these vehicles are gradually becoming attractive targets for cyberattacks. This study proposes the development of an efficient platform based on the Message Queuing Telemetry Transport (MQTT) protocol for UAV control and Denial-of-Service (DoS) detection embedded in the UAV system. For the efficiency test, latency, network and memory consumption on the platform were measured, in addition to the correlation between payload and delay time. The results of efficiency tests were collected for the three levels of quality of service (QoS). A strong correlation greater than 90% was found between delay and data size for all QoS levels, showing almost a linear proportion. In DoS detection, the best results were a true positive rate (TPR) of 0.97 with 16 features from the AWID2 dataset using LightGBM with Bayesian optimization and data balancing. Unlike other studies, the built platform shows efficiency for UAV control and guarantees security in the communication with the broker and in the Wi-Fi UAV network.

## 1. Introduction

The Internet of Flying Things (IoFT) is a new research domain that aims to integrate unmanned aerial vehicles (UAVs) to the Internet of Things (IoT) to support different applications, and it has been receiving increasing attention in both civilian and military research recently [1]. UAVs represent a worldwide known term that encompasses all types of autonomous, semi-autonomous and remote-controlled aircraft. Those vehicles are also known as drones in some contexts. UAV applications have become very popular, among others, in the military, industrial control, and agriculture. For example, there are over 40 countries using UAVs for military operations [2]. The popularization of UAVs has been maintained due to their dynamic reconfigurability, response agility, and facilitated deployment. Additionally, UAVs have benefited from many technological advancements in portable battery supply, portability, sensor size, weight reduction, and advancements in Global Positioning System (GPS) and communication technologies.

Many areas can benefit from IoFT due to its increasing notoriety over the last few years, such as emergency assistance for victims rescue and tracking; smart cities for infrastructure and traffic flow monitoring; industry and commerce for delivery and factory supervision; smart farms for chemical spraying; environment for forest fires and illegal activity monitoring; and others [3]. There are a few companies already utilizing UAVs in their operations. One example is Amazon, with their aerial and autonomous delivery of goods to customers [4].

In order to continuously exchange data without compromising the security or reliability of the information, strategies for the communication between UAVs and the operations teams must be adapted and work with mobile connection protocols, such as General Packet Radio Service (GPRS), Global System for Mobile Communications (GSM), Wireless Fidelity (Wi-Fi), 4G, 5G, Sigfox, and others, considering that the flight area of a UAV may be very large. However, there are many dangers and limiting factors for UAVs and IoT applications, such as resource constraints, signal collision and interference, infrastructure and other details [1]. For example, network security has become a critical factor with the rapid growth in the IoT around the world and, because of the relative ease and low cost, cyberattacks on UAVs are becoming popular very quickly [5]. One of the common attacks in UAVs is Denial-of-Service (DoS), which interrupts communication overloading the network with large volumes of false data [6], bringing risks to people close to the vehicle, as well as damage and possible loss of the UAV. Machine learning (ML) is one of the techniques applied to detect wireless network attacks, including DoS attacks, where algorithms such as random forest (RF) and light gradient boosting machine (LightGBM) are commonly used [7,8]. Other examples of studies that use ML concepts in their attack detection strategies are [9,10,11]. The advantage of a partial way to obtain logical, applicable, reproducible and evaluable conclusions makes ML an interesting tool for attack detection.

This paper proposes the utilization of the MQTT (Message Queuing Telemetry Transport) protocol for developing a UAV remote control platform, a light, power, and efficient solution for information exchange. That way, it is expected to develop a low latency and low memory communication channel, attesting to its functionality by virtual tests and analysis. The developed platform is tested with a real UAV, where a microcontroller with access to the MQTT broker is connected to the UAV’s wireless network. Authentication and Secure Sockets Layer/Transport Layer Security (SSL/TLS) certificates are used to communicate with the broker to ensure platform security. In the wireless network, a ML model is built to detect DoS attacks on the UAV network, based on the Aegean Wi-Fi Intrusion Dataset 2 (AWID2). Therefore, the contributions in this work are:

- We develop an MQTT-based platform for UAV control that tests latency, network and memory, and measures the correlation between payload and delay time;
- We implement authentication and SSL/TLS certificates in communication with the broker server. From this, there is a guarantee in the communication between controller and UAV on the Internet;
- We build an ML model to detect DoS attacks on the UAV network, implementing algorithms based on decision trees with the gradient boosting technique. In addition, data balancing and Bayesian optimization in LightGBM are applied.

This paper is organized as follows: Section 2 presents and reviews background concepts with further detail; Section 3 details the platform development and efficiency tests in MQTT, as well as the DoS detection model design; Section 4 presents the results of the MQTT efficiency for UAV control and the DoS detection model; finally, Section 5 concludes the paper and presents future work.

## 2. Background and Related Work

This section presents background information and related work. It discusses MQTT protocol and presents some possible cyberattacks in UAVs. This section also presents related work regarding two topics: platforms for UAV/robot control and wireless network attacks detection on IoT devices.

### 2.1. Broker and the MQTT Communication Protocol

**Figure 1:**
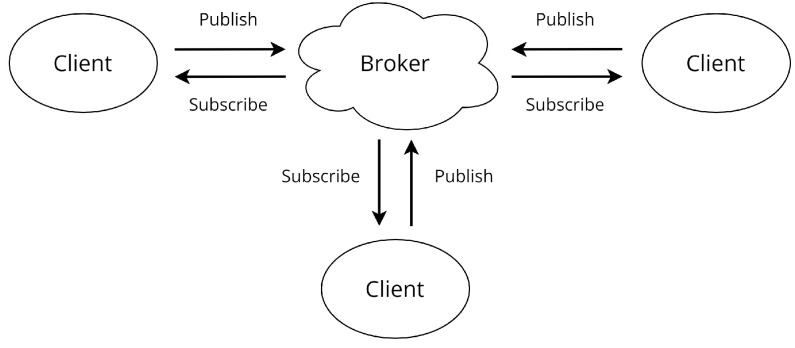
Representation of MQTT connection.

**Figure 2:**
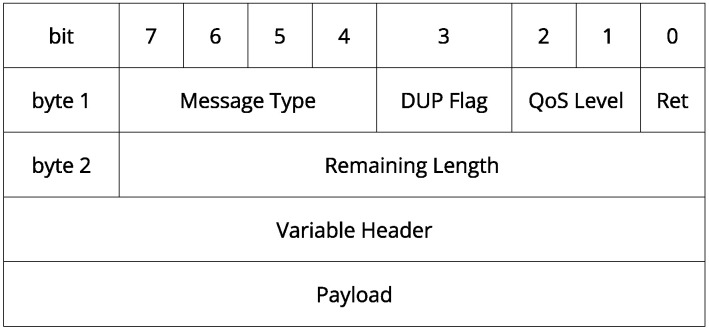
MQTT header.

MQTT is a communication protocol based on a publish/subscribe model, allowing a client to send messages to all other clients subscribed on the same topic [12], as presented in Figure 1. It works on top of the TCP/IP with light transport overhead (Figure 2) and other minimizations that makes MQTT ideal for machine-to-machine (M2M) and IoT applications [12,13]. Quality of service (QoS) defines a set of technologies that aims to give its user better assurance of message delivery, guaranteeing high reliability and priority in the communication task. The MQTT protocol has three levels of QoS [12,13], defined below:

- QoS level 0 (At most once): Message delivery is carried out based on the operational conditions without any kind of confirmation. Communication tasks send the message only once, without any kind of feedback (losses may occur). This mode allows for faster message transfer at the cost of security;
- QoS level 1 (At least once): The message is delivered and the sender requests an acknowledgment of arrival from the receiver, known as PUBBACK. If the receiver does not provide this feedback, the message is re-sent (double arrivals may occur);
- QoS level 2 (Exactly once): The messages are delivered only once, backed by a four-way handshake system. The message stay stored at the sender and receiver until it is completely processed.

The broker handles the communication between clients, being responsible for the message authentication, delivery, and confirmation. The memory and processing capacity will be on the cloud, decreasing the required resource on the devices (clients).

An open source, light, easy, and secure MQTT broker is Mosquitto. It supports user and password configuration for client connections and SSL/TLS certificates for encrypting the connection [14]. The open-source Paho library could be employed for the client-broker communication and implemented in languages such as Python, C, and Java [15].

### 2.2. Cyberattacks on UAVs

Cyberattacks on UAVs have become increasingly more popular in recent years because of their ease and low-cost [16], with the limited computing power and communication capability being strong candidates for their vulnerabilities [11]. Attacks that lead to loss of service availability, also known as DoS, are common in UAVs due to their ease and are considered trivial in versions of the standard up to 802.11n, due to non-protection of management messages. Some of these attacks are presented below, which were carried out to build the AWID2 dataset [17]:

- Deauthentication: A simple and efficient attack on Wi-Fi networks. Deauthentication packets are transmitted unprotected and spoofed by the attacker. Upon receiving the packets, the client leaves the network instantly without further action;
- Authentication Request: As the maximum number of clients that can be kept in the access point (AP) client association table is limited, the attacker exhausts the AP’s resources by overflowing the table;
- Beacon: This type of attack can be carried out in two ways: (1) the attacker transmits a constant stream of false beacons that advertise non-existent extended service set identifiers (ESSIDs); and (2) the attacker transmits a flood of spoofed beacon frames with a specific ESSID that matches different basic service set identifiers (BSSIDs);
- Probe Response: The attacker monitors probe request messages from valid clients, and acting as an AP, a flood of inaccurate and fake probe responses are transmitted to the stations (STAs);
- Clear to Send (CTS): In this flooding attack, the attacker constantly transmits CTS frames to itself or to another STA, which forces the rest of the STAs on the network to continually postpone transmission.

### 2.3. Related Work

There are security and privacy challenges that exist in the exchange of information between UAV communications for the purposes of attacks and espionage. This affirms the importance of research in the area and the diverse needs in control platforms [1]. Therefore, the related work are presented here, which are divided into two categories: a platform for controlling UAVs and robots, and the detection of wireless network attacks on IoT devices.

#### 2.3.1. Control Platform

Hillar [18] proposed a UAV control system with MQTT programmed in Python that has authentication and Transport Layer Security (TLS) encryption. It defines exclusive topics for each vehicle and other topics to confirm the command execution. The messages are JavaScript Object Notation (JSON), containing their commands and respective variables. The tests were applied in IoT-focused boards, executing the Paho Python library and level 2 QoS. Giusti et al. [19] built a robot interaction system based on MQTT, a web interface for control and a Raspberry Pi 3. With the Amazon Elastic Compute Cloud (EC2), the authors made the nodes communicate and, virtually, demonstrated them working. Lee et al. [20] correlated the packet loss delay in their work, considering three different QoS levels for the MQTT protocol in a connected and wireless scenario with 3G technology. Çorak et al. [21] proposed a solution with a new platform based on a combination of the methods presented in the MQTT and the Micro Air Vehicle Link (MAVLink) protocols. The presented solution combines both advantages, resulting in a significant gain in its efficiency. Chen et al. [22] opened a discussion about the aspects and challenges of security criteria for applications with the MQTT protocol, promoting an ample revision study of the protocol, considering different scenarios, possibilities, and types of attacks. Finally, Rodrigues and Branco [23] presented the Cloud-SPHERE platform as a secure approach to UAVs connections. This platform was structured from the HAMSTER architecture that was designed to allow adaptability to different vehicles. The solution adapts the architecture with a flexible, heterogeneous, and secure platform that operates in four different scenarios and promises to cover the most different needs.

From the studies, it is noted that, generally, the existing works focus more on efficiency [18,20,21] or on security [22,23], and there are not enough MQTT tests to control UAVs, or, that is, perform the latency, network and memory tests. For example, in Lee et al. [20], there is a study of the correlation of packet loss delay in the three levels of QoS, but the authors did not implement encryption or impact tests for each level in terms of package and bit quantities for a determined load. To ensure security in communication with the broker, works usually use SSL/TLS certificates and authentication [18,21].

#### 2.3.2. Detection of Wireless Network Attacks

In related work, three studies were raised that used the AWID2 dataset to detect cyberattack behavior, represented by the references [7,8,9]. Rezvy et al. [9], in their work, used a dense neural network to detect attacks on 5G networks. The proposed model used supervised and unsupervised tests, and from the applied tests, it achieved a high detection rate for flooding, impersonation and injection attacks. In Bhandari et al. [7], the authors concluded that the RF, extreme gradient boosting (XGBoost), LightGBM and categorical boosting (CatBoost) algorithms have similar results in terms of accuracy, precision and recall when applied to a reduced subset of features selected by the SHapley Additive exPlanations (SHAP) method, in addition to being executed in a reduced time. Chatzoglou et al. [8] conducted a survey to identify how many features are needed to obtain an optimized ranking in attack detection based on ML, obtaining positive results with a total of 16 features.

In the study by Bouhamed et al. [24], a lightweight intrusion detection and prevention system (IDPS) module for UAVs was proposed, which is trained by applying deep reinforcement learning (DRL), specifically deep Q-learning (DQN), allowing UAVs to automatically identify suspicious activity and take action. In Ahmed et al. [10], an anomaly detection system was built using unsupervised learning. Data are collected from network traffic by performing deauthentication and WPA2 cracking attacks. Lastly, Al-Haija et al. [11] used deep convolutional neural networks (UAV-IDS-ConvNet) to detect cyberattacks considering UAV platforms in their tests, obtaining efficient results with a two-class classifier.

Regarding the detection of attacks on the wireless network, the works use unsupervised [9,10] and supervised [7,8,9,11,24] approaches, where interesting results are obtained using the algorithms XGBoost, LightGBM and CatBoost with a reduced set of features [7,8], where simple ML models obtain comparable results with deep neural networks (DNN), such as deep reinforcement learning (DRL) [24] and convolutional neural networks (CNN) [11]. In the study by Chatzoglou et al. [8], LightGBM obtained the best F1 score in the AWID2 dataset, being better than DNN. Detected attacks usually include flooding, impersonation and injection attacks [7,8,9], or deauthentication and WPA2 cracking attacks [10]. It is observed that the works that detect attacks are not integrated into platforms, and generally, the ML models are multiclass; there is no specific model to detect DoS. Thus, a platform is needed that integrates efficiency and security, encompassing features of the aforementioned platforms and integrating a model for the detection of UAV attacks.

## 3. Materials and Methods

This section presents the MQTT-based UAV control platform developed in this work. Its objective is to test the communication efficiency between the controller and the UAV and to construct a predictive model for attack detection in the wireless network between the UAV and a Raspberry Pi board.

The broker server was installed in a Linux environment from the infrastructure of the University of São Paulo. The open source project Eclipse Mosquitto [14] was used in the platform for the MQTT communication due to being lightweight, easy to implement, and having support for security mechanisms, allowing the configuration of a username and password for client connection. To send and receive messages (the MQTT client implementation), the Eclipse Paho library was used, with the implementation made in Python. The Mosquitto Python Client project was donated to Eclipse Paho in June 2013 and allows integration with several programming languages, such as C or Java [15].

The security in the MQTT communication is guaranteed by the use of a username and password for access to the broker server and TLS certificates to encrypt and authenticate the connection, ensuring the confidentiality of the exchanged data, similar to the Hillar proposal [18]. These certificates used the X.509 Public Key Infrastructure (PKI) standard and were self-signed with an SHA-256 signature hash through OpenSSL.

**Figure 3:**
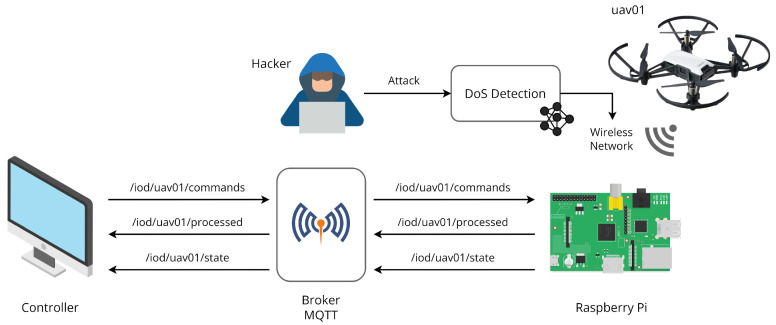
Platform overview.

Figure 3 shows the platform overview. The Raspberry Pi Model B was connected to the access point generated by the UAV. This model contains an Ethernet port and allows the connection of a Wi-Fi dongle to connect to the UAV. To ensure the Wi-Fi connection security between the microcontroller and the UAV, a ML model for DoS detection was built. Three topics were generated for the communication between the controller and the Raspberry Pi. The topic structures are similar to Hillar’s work [18], as shown:

- /iod/uav01/commands: for sending the commands;
- /iod/uav01/processed: for sending the commands execution confirmation;
- /iod/uav01/state: for sending the UAV current status.

Commands are structured in a JSON string containing a timestamp, the action required, and a value for the command. This last value describes the metric to be executed such as the distance for moving forward or the angle for the rotation. To take off, for example, the message would be: “{“time”: “2022-05-10 16:23:25:272750”, “action”: “takeoff”, “value”: 0}”. About the UAV actions, the following commands have been implemented in the platform, where value is a positive integer defined by the controller: takeoff; land; up value; down value; forward value; backward value; left value; right value; rotate_clockwise value; and rotate_counterclockwise value.

**Figure 4:**
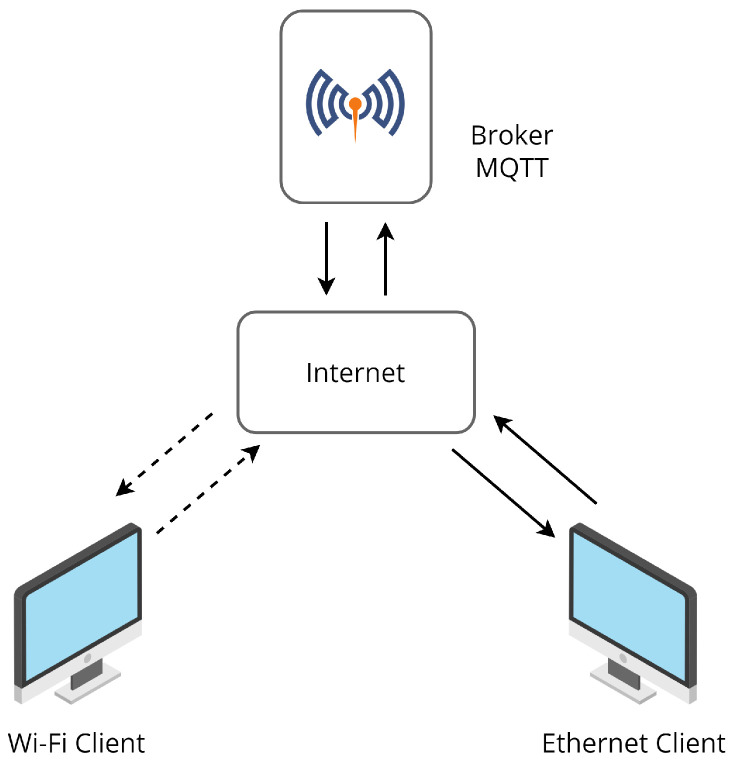
Infrastructure of the connection.

All the programs and algorithms for testing the platform efficiency, except for the broker server, were run in a local laptop with Windows 10, 64 bits, 16 GB of RAM, and an Intel Core i7-6500U processor. It was located 450 km away from the broker. For the wireless tests, it was connected to a Wi-Fi router at a distance of 1 m. Figure 4 shows the infrastructure of the connection. The Google Collaborary (Colab) platform was used in its free version to train, test, and validate the DoS detection model.

Regarding the “state” topic, coordinates were simulated for the efficiency test. However, when connecting the platform with a real UAV, the data sent to the controller are the angles of pitch, yaw and roll, speed on each axis, mean temperature, battery level, and height. These data are collected on account of using the DJI Tello, which consists of an extremely light UAV with only 80 g counting the propellers and battery, making this module low cost and portable. In the future, other UAV models, such as the Parrot Bebop 2 and DJI Spark, will be added.

Thus, the platform allows the use of real or simulated UAVs. In the efficiency tests, the simulated UAV was used, since the efficiency is measured only up to the Raspberry Pi connection. After completing the efficiency tests, the platform was tested with DJI Tello to see how it works in practice.

**Figure 5:**
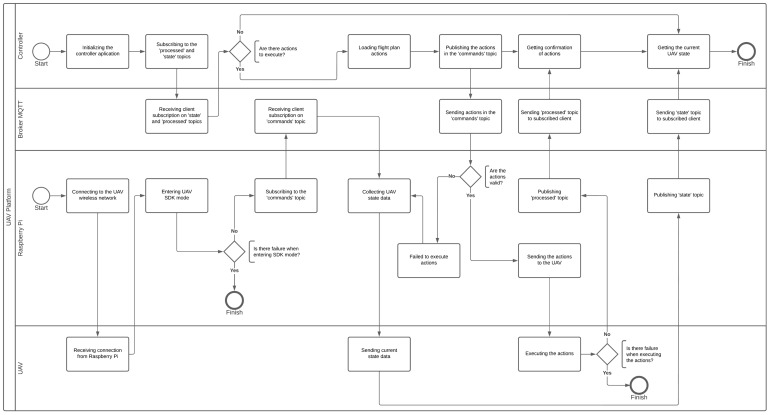
Platform BPMN flowchart.

As a way to better detail the entire functioning of the platform and how each component communicates, a BPMN flowchart is illustrated in Figure 5. Regarding the flowchart, initially the Raspberry Pi connects to the UAV network, and after that it enters SDK mode. If there is no failure to enter SDK mode, the Raspberry Pi subscribes to the “commands” topic to be able to receive controller actions, and the request is received by the MQTT broker. After that, the board starts collecting data from the UAV and publishing it to the controller through the “state” topic. On the other hand, the controller initializes the application and subscribes to the “processed” and “state” topics to receive command confirmation and the state of the UAV, respectively, the requests for which are received by the broker. After that, the platform checks if the controller has actions to execute, and if there are no actions, the controller receives the current state of the UAV. If there are actions, a flight plan is loaded and each action is published in the “commands” topic, and then the controller receives confirmations if no errors occur. When the Raspberry Pi receives the commands, there is validation, and if they are valid, the actions are sent and executed in the UAV. The vehicle performs the actions if no failure occurs, and the confirmation is sent to the controller.

Therefore, with the development of the platform, it is possible to obtain two contributions: latency, network and memory tests using MQTT for UAV control, in addition to the detection of DoS attacks on the Wi-Fi network generated by the UAV, as detailed by the following subsections.

### 3.1. Platform Latency, Network and Memory Tests

Latency was measured using two Python codes, one that sends and another that receives a message in each QoS level with a timestamp, obtaining the delay needed to send and receive the payload. This message also contained a JSON string of data to simulate GPS coordinates. In this way, the final string obtained the following layout: “{“time”: 2022-06-07 12:58:08:122377, “data”: “000 000 000 000 000 000 000”}”. The delay was determined through the arithmetic average of 50 samples for each QoS level. Packets were collected through the software Wireshark, obtaining their numbers and sizes (bytes). This information was collected for each level of QoS for which the latency tests were conducted.

The memory (RAM) was measured using the Python module memory-profiler that obtains the memory in MebiByte (MiB) needed for running a specific Python program. This test is relevant to evaluate the memory needed to encrypt and decrypt the messages and the suitability to an embedded system. The graph of consumption was plotted after the platform sent 150 telemetry payload samples in different QoS levels; in this way, it simulated the memory that would be used by a UAV running the proposed platform.

### 3.2. Correlation between the Payload and the Delay Time Test

For determining the correlation between the payload and the delay needed to send and receive the data, two Python codes were used: one to send and another to receive messages. The payload size was varied from 1 kB to 10 kB, with a difference of 1 kB for each measurement, and from 10 kB to 100 kB, with a difference of 10 kB for each measurement. The final value was the arithmetic average of 50 samples taken for each measurement. These samples were collected for all the three QoS levels, so it would be possible to calculate the correlation for each level. To calculate the correlation between payload and delay time, Equation (1) was used.

The factor that scales *X* to produce *Y* is denoted by (1), where cov(X,Y) is the covariance of *X* and *Y*, $S_{x}$ is the standard deviation of *X* and $S_{y}$ is the standard deviation of *Y*. It is called the Pearson correlation coefficient (*r*), varying from −1 to 1 [25]. When *X* (payload) and *Y* (delay) have r≠0, then *X* and *Y* are said to be correlated, and when it is zero (r=0), the random variables are said to be uncorrelated. If r>0.7, it is considered a strong correlation.

(1) $$r=\frac{cov(X,Y)}{S_{x}\cdot S_{y}}=\frac{\sum_{i=1}^{n}\left(X_{i}-\bar{X}\right)\cdot \left(Y_{i}-\bar{Y}\right)}{\left(n-1\right)\cdot S_{x}\cdot S_{y}}$$

### 3.3. DoS Detection in Wireless Network

**Figure 6:**
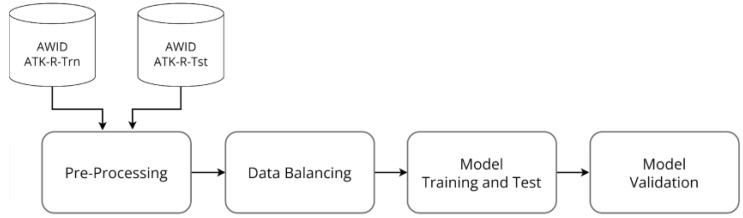
ML pipeline.

Since the connection between the Raspberry Pi and the UAV is accomplished through a wireless network, a predictive model is trained to detect DoS attacks in this network. For this, the AWID2 dataset was applied, which used a physical laboratory to emulate a typical Small Office/Home Office (SOHO) network infrastructure, with devices connected in a wireless network. Then, various types of attacks were carried out, dividing them into three main classes: flooding, impersonation and injection [17]. Thus, it was necessary to pre-process the dataset and balance classes, and then train/test the model and validate it. All these steps are illustrated in the ML pipeline in Figure 6. In the following, each of the steps is detailed.

#### 3.3.1. Pre-Processing

For the construction of the model of this work, only flooding attacks (also known as DoS) were considered, including deauthentication, disassociation, authentication request, fake power saving, clear to send (CTS), request to send (RTS), beacon, probe request, and probe response attacks. The AWID2 has a division of datasets into training (Trn) and test (Tst), with a full (F) and reduced (R) version. Since the full version has more than 108 h of data collected and this work uses the Google Collab tool, the reduced version was selected, which has 1 and 1/3 h of network traffic collected for training (AWID-ATK-R-Trn) and test (AWID-ATK-R-Tst), respectively [17]. The AWID2 dataset was selected because it deals exclusively with wireless network attacks and is well consolidated for intrusion detection [8,9].

In this way, the training and test datasets were grouped because there are attacks in the test that are not present in the training dataset. Only 16 features were selected from the dataset based on the results from Chatzoglou et al. [8], in which those features were enough to achieve a high score. Table 1 presents each of the features, their descriptions and types. Only float and categorical types were used, and ML algorithms with support for these data types were selected.

After selecting the features, the null rows and constant column (radiotap.present.tsft from Chatzoglou et al. [8]) of the dataset were removed. The constant column was excluded because it works only with data from the normal or DoS class. Min-max scaling was applied for float data, and one-hot encoding (OHE) was not applied to categorical data because the ML algorithms used work directly with this type, which already benefits the process. The ATK version of the dataset was used, which presents each DoS attack, that is, multiclass, the attacks were equally divided. After that, all attack classes were converted to the DoS class. The number of samples obtained after pre-processing is shown below:

- Training and Test Dataset: 1,663,689 total samples, being divided into 1,621,257 in the normal and 42,432 in the DoS class. These samples were used for model training, so data balancing was required, which is described in Section 3.3.2;
- Validation Dataset: 554,568 total samples, being divided into 540,419 in the normal and 14,149 in the DoS class. As these samples were used for model validation, it was not necessary to perform data balancing.

In addition to using all the previous features, the dataset was divided into a set of columns to build other models to see how much it impacts the result. For this, four sets were defined based on Chatzoglou et al. [8], containing the three columns of type float (frame.len, adiotap.dbm_antsignal and wlan.duration), and one more categorical column. For Set 1, the categorical column used was “wlan.fc.subtype”; in Set 2, the categorical column used was “wlan.fc.type”; in Set 3, the categorical column used was “wlan.fc.ds”, and finally in Set 4, the categorical column used was “wlan.fc.protected”. The objective is to test how much information is kept with a quartet of features.

#### 3.3.2. Data Balacing

For the balancing of training and test data, the random undersampling (RUS) technique is applied. The RUS consists of a non-heuristic method that aims to balance the data through random elimination from the majority class. Despite being simple, the RUS has great results with a low response time [26]. After data balancing, 42,432 samples are of the normal and 42,432 of the DoS class. Therefore, the bias of ML algorithms towards the majority class is avoided, which would result in a misclassification rate of data that are from minority classes.

#### 3.3.3. Model Training and Test

For the model training, ML algorithms based on decision trees with the gradient boosting technique were applied. These algorithms are XGBoost, CatBoost, and LightGBM. XGBoost gained prominence due to its high scalability in all scenarios, which allows its execution from a common computer to distributed configurations [27]. CatBoost has great quality without parameter adjustment, supports categorical features, has a fast and scalable GPU version, and has improved accuracy [28]. Finally, LightGBM was designed to have high efficiency and speed, low memory usage, better accuracy, support for GPU and parallel learning, and optimizations during tree construction. The hyperparameters used in the algorithms are the default of their respective libraries in the Python language. However, in the LightGBM algorithm, training is also performed with selection by Bayesian optimization, which finds the global minimum of a function with the least number of iterations. It is important to note that the XGBoost and LightGBM algorithms natively support categorical features in the Python library.

For testing, in addition to avoiding the occurrence of overfitting and underfitting, the stratified *k*-fold cross-validation was used. In this technique, there are *k* iterations in the dataset. At each iteration, the dataset is divided into *k* folds, where training is performed on k−1 folds, and testing is carried out on the fold not used by training. With stratification, the proportion present in the dataset is maintained in the division of each of the folds [29]. The value k=5 was chosen due to the size of the dataset, being an interesting value for analyzing the behavior of the model without increasing the computational cost [30]. The evaluation metric used in the model is the area under the ROC curve (AUC), F1 score and training time. The training and test process is the same for all columns and each column set.

To evaluate the ML model, precision, recall, F1 score, and accuracy are extracted from the confusion matrix. The confusion matrix is a table used to verify the model performance, defining the following terms: true positives (TP) and true negatives (TN), which are the DoS and normal samples correctly identified, respectively; false negatives (FN) are DoS samples incorrectly classified as normal; and finally, false positives (FP) are normal samples classified as DoS. Based on this, accuracy (2) is the fraction of the predictions that the model was right. Precision (3) is the metric responsible for answering which proportion of positive identifications is correct. Recall (4) answers which proportion of actual positives was correctly identified. F1 score (5) combines accuracy and recall, where harmonics are performed between the two.

(2) $$\mathrm{Accuracy}=\frac{TP+TN}{TP+TN+FP+FN}$$

(3) $$\mathrm{Precision}=\frac{TP}{TP+FP}$$

(4) $$\mathrm{Recall}=\frac{TP}{TP+FN}$$

(5) $$F1\;\mathrm{score}=\frac{2\cdot \mathrm{Recall}\cdot \mathrm{Precision}}{\mathrm{Recall}+\mathrm{Precision}}$$

In addition to the metrics extracted from the confusion matrix, there is the receiver operating characteristic (ROC) curve, composed of two axes, where on the x-axis, there is the false positive rate (FPR), and on the y-axis is the true positive rate (TPR). The FPR is calculated based on (6) and indicates the rate of positive samples that were misclassified. On the contrary, the TPR, called recall, is calculated based on (4), as presented above. To understand the ROC curve more easily, AUC is used, which is the area under the curve, as explained before, and its value varies in the range of [0.0; 1.0].

(6) $$\mathrm{FPR}=\frac{FP}{FP+TN}$$

#### 3.3.4. Model Validation

After training and testing, models built with LightGBM with Bayesian optimization are tested in the validation dataset. Thus, the true positive rate (TPR) and false positive rate (FPR) of the models are obtained using all columns and Set 1, Set 2, Set 3 and Set 4. From this, it is possible to know which models are the most reliable and applicable in practice. If any hacker tries to carry out any of the attacks included in the model, the system will notify the controller.

## 4. Results and Discussion

This section first presents the results of latency, network, and memory tests on the platform and the correlation between payload and delay time. After that, the performance of the models to detect DoS attacks on the wireless network is shown in both training and test, as well as the validation with new data.

### 4.1. Platform Latency, Network and Memory Results

**Figure 7:**
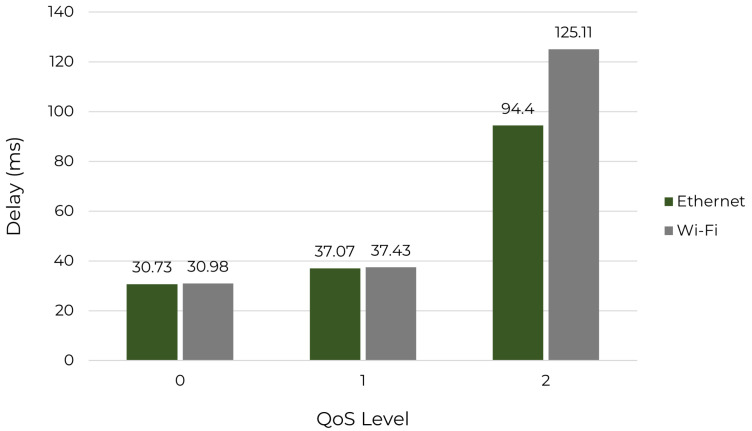
Delay measured for Ethernet and Wi-Fi connections.

The delay for sending a default telemetry payload was measured for Ethernet and Wi-Fi connections. The data collected on an Ethernet connection are reference values for later comparison. Figure 7 shows the results for latency for every QoS option available.

According to Figure 7, QoS level 1 has 20.6% more delay than QoS level 0, though the numeric difference is only 7 ms. For QoS level 2, the delay on Ethernet is 154.7% greater than the delay on QoS level 1 and 207.2% greater than the delay on QoS level 0, showing a numeric difference of, respectively, 57.33 ms and 63.67 ms. Comparing the delay of QoS level 2 on Ethernet and on a Wi-Fi connection, it is noticed that Wi-Fi is 32.5% greater than on Ethernet. However, for QoS level 0 and level 1, the values are almost the same (less than 1% of difference). These results were expected because more reliability requires more messages exchanged before the payload is confirmed.

The packets and their size in bytes were collected for some tasks on Ethernet and Wi-Fi connections, and the results are shown in Table 2. These tasks simulate actions that could be performed in a real scenario through the platform.

As shown in Table 2, the data consumption tends to be similar on Ethernet and Wi-Fi, varying less than 3% for all tasks. The packets’ quantity and size in bytes increase according to the level of QoS used, varying 33.9% between QoS 0 and QoS 1, 53.0% between QoS 1 and QoS 2 and 104.9% between QoS 0 and QoS 2 (for sending a telemetry payload). Analyzing the data consumed when receiving a payload containing a command, QoS level 1 is 40.9% greater than level 0, and QoS level 2 is 71.6% greater than level 1 and 141.8% greater than level 0. Similar to the delay results, the difference in bytes consumed is explained by the need to send more messages when increasing the QoS level.

**Figure 8:**
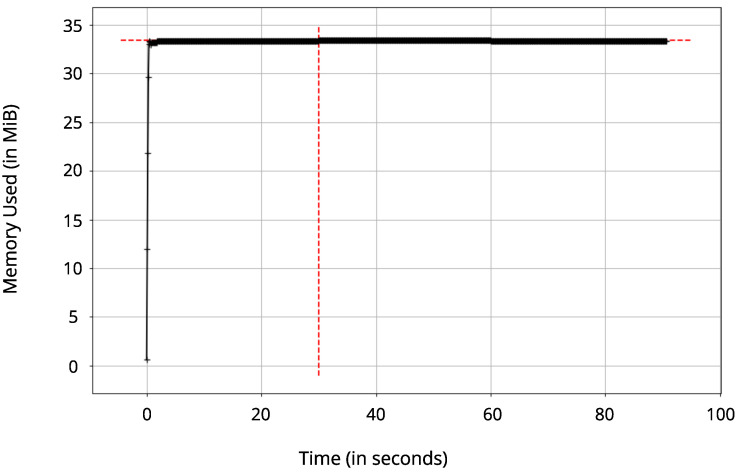
Memory consumed as a function of time when running the platform, where the average consumption is 34.33 MiB.

Figure 8 contains the memory consumed in MebiBytes (MiB) when running the platform. Converting the memory used per MegaByte (MB), a value of about 36 MB is reached. Thus, the platform did not require much RAM resources, being suitable for implementation in an embedded system. Probably, when implementing the algorithms in C, the RAM required would be lower than when using Python. However, for applications with higher processing power, such as Raspberry Pi boards, Python code can be used given its better scalability and ease.

### 4.2. Correlation between the Payload and the Delay Time Results

**Figure 9:**
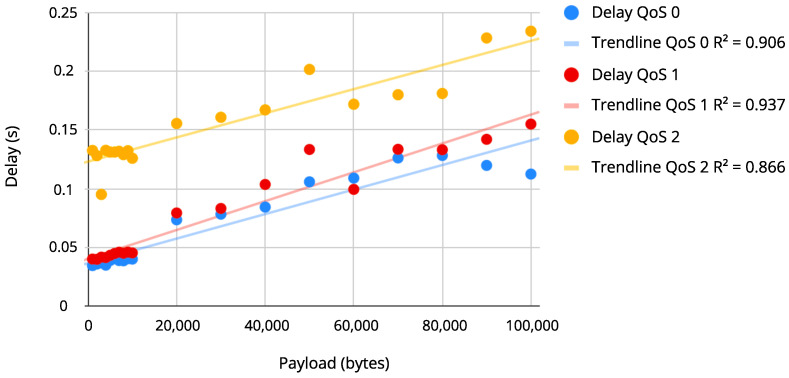
Payload × Delay on Wi-Fi connection.

**Figure 10:**
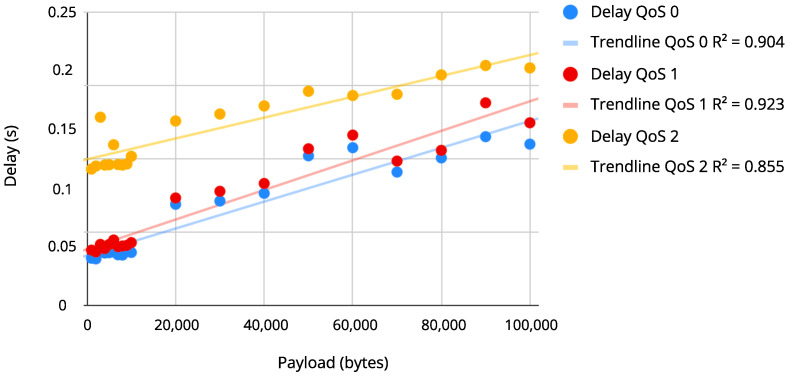
Payload × Delay on Ethernet connection.

After measuring the delay for different payload sizes in each level of QoS, two graphs were plotted to show the distribution of these values, as seen in Figure 9 and Figure 10. The correlation analysis showed how the two variables are related by a correlation coefficient that allows to predict the value of one variable depending on the value of the other (packet size and delay). To perform the correlation, the data were measured under similar conditions on Wi-Fi and Ethernet connections for the three QoS levels. Figure 9 and Figure 10 represent the scatter diagram for the payload and delay variables on Wi-Fi and Ethernet, respectively, and suggests how these variables are correlated and the direction of the correlation (what happens to the delay values when payload values increase or decrease). In Figure 9 and Figure 10, it is possible to observe that there is a linear correlation between the variables, i.e., the delay values increase as the payload size increases. The relation strength can be calculated through the correlation as in Equation (1), indicating a strong positive correlation between delay and payload.

The correlation (*r*) between the delay and the size of the payload is shown in Table 3. It is possible to verify that the correlations were considered strong for all three levels of QoS. The QoS level 1 presented the strongest correlation between delay and payload on an Ethernet connection, and QoS level 0 presented the strongest correlation on a Wi-Fi connection. The strongest correlations are highlighted in bold for better visualization.

Besides the *r* coefficient, Table 3 also shows the coefficient of determination ($R^{2}$). This coefficient describes the proportion of the average variation of the delay explained by the payload size. The greatest $R^{2}$ found was 0.937 for QoS level 1 (Ethernet); that is, more than 93.7% of the delay average variation is explained by the payload size variation.

### 4.3. DoS Detection Results

This section first compares the XGBoost, CatBoost, and LightGBM (with and without Bayesian optimization) algorithms in training and testing with all dataset columns in terms of AUC and training time. Then, it presents the application of LightGBM with Bayesian optimization on each set of columns, which were defined in Section 3.3.1. Finally, the test results of the models built on the validation dataset are shown, allowing to verify the most reliable model and to assess the necessity of all columns.

#### 4.3.1. Model Training and Test Results

**Figure 11:**
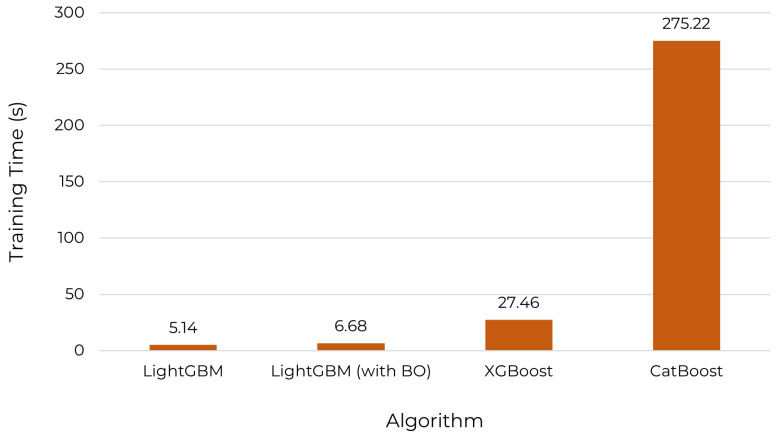
Algorithm training time.

Initially, the three algorithms were used with default hyperparameters available in their respective libraries; after that, Bayesian optimization (BO) was applied in the LightGBM algorithm with 50 initial points and 500 iterations. The optimized hyperparameters were: lambda_l1; lambda_2; num_leaves; feature_fraction; bagging_fraction; bagging_freq; and min_child_samples. The stratified cross-validation technique with k=5 was applied in the training and testing of the algorithms. Table 4 shows a comparison of each algorithm in training in terms of AUC. The training time in seconds, which is the average of three training times, is illustrated in Figure 11. The F1 score was not presented in the table because for all algorithms, its value was 0.99.

It can be seen from Table 4 and Figure 11 that all algorithms had high AUC, but the biggest highlight is for LightGBM, which had the highest AUC and the shortest training time. It is observed that the training time did not increase much with the use of Bayesian optimization, taking approximately 7 s. XGBoost and CatBoost were excellent as well, with the only issue being training time. For example, CatBoost took 275 s to perform the training, where one of the problems for the slowness can be the identification of categorical data. Because of the higher AUC of 0.9983 and the low training time, LightGBM was selected for training the dataset with each column set.

When training and testing the model with Set 1, Set 2, Set 3 and Set 4 of columns, the results regarding AUC and F1 score are shown in Table 5. As explained before, in each training, LightGBM was applied with the hyperparameters selected with Bayesian optimization. From the performance in each set, it is noted that with Set 1, there was a low decrease in relation to the result with all columns. However, in Set 2, Set 3 and Set 4, the decrease was much greater, mainly on the F1 score metric. With this, it is possible to conclude that the categorical column present in Set 1, which is “wlan.fc.subtype”, as already mentioned in Section 3.3.1, is relevant for the detection of DoS attacks.

Comparing the results of this work with three other related works that use the AWID2 dataset, Rezvy et al. [9] obtained an accuracy of 0.998 for flooding-, injection-, and impersonation-type attacks using a autoencoded DNN with 36 dataset features. In Bhandari et al. [7], the authors achieved an F1 score of 0.9997 with random forest with a reduced set of features using the SHAP method. Finally, Chatzoglou et al. [8] achieved an F1 score of 0.9536 in identifying attacks using LightGBM. In addition, the features were studied by experts in this work, defining the 16 most relevant features to detect attacks. This work differs from Rezvy et al. [9], Bhandari et al. [7], and Chatzoglou et al. [8] by proposing the binary identification of attack, that is, we use only the normal classes and DoS attack, also known as flooding attack. Furthermore, Bayesian optimization is applied in LightGBM, and the focus is unique to UAV.

#### 4.3.2. Model Validation Results

**Figure 12:**
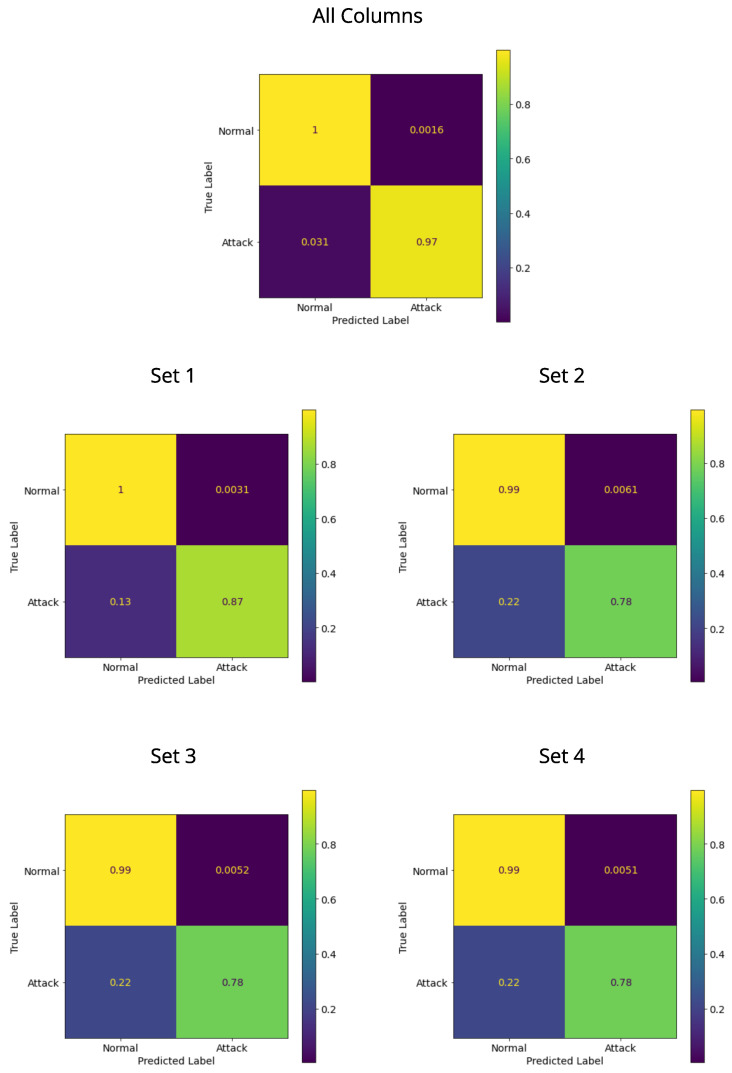
Confusion matrices with validation dataset.

Figure 12 shows the results for testing the models with all columns and sets in the validation dataset. LightGBM with Bayesian optimization was applied in the training of each model. Of the confusion matrices, the best model was using all columns, where the model obtained a TPR of 0.97, corresponding to how many of the DoS samples were correctly classified, and an FPR of 0.0016, which designates the number of DoS samples misclassified. The model built only using Set 1 of the columns obtained a TPR of 0.87 and FPR of 0.0031. There is a decrease in performance compared to the previous model; however, it is still acceptable because it uses only one categorical column. The other models generated with Set 2, Set 3 and Set 4 of columns decreased even more, with FPR ranging between 0.0051 and 0.0061 and a TPR of 0.78.

Therefore, with the validation of the models, it is possible to confirm the training and test results, where the models with all columns and Set 1 were the best. Since the model is for IoT devices, a lightweight classifier is important due to constrained resources, so making use of only Set 1 is an option to consider.

## 5. Conclusions

In this paper, a UAV control platform was presented and proved itself effective in sending and receiving messages, attesting to the hypothesis of a lightweight, secure, and reliable data exchange environment based on MQTT. In addition, a model to detect DoS attacks on the UAV network was built, obtaining interesting results in the validation step. Thus, this work has the differential of uniting efficiency and security in a platform to control UAVs, guaranteeing security in the communication with the broker with authentication and SSL/TLS certificates, and in the UAV network with the microcontroller applying ML techniques.

The latency between the two ends was low for QoS level 0 and QoS level 1, and due to the fact that the messages contain a timestamp string (each payload is unique), QoS level 1 would be ideal for a real scenario, confirming the delivery of the payload at least once. Analyzing the packets and its lengths in bytes to perform some tasks, QoS level 1 showed to be a good choice for sending and receiving payloads, having a size of less than 330 kB for these tasks. Memory usage also had a low value of about 36 MB, making it possible to use the platform on an embedded Linux system.

The calculated correlation between the payload size and the delay was over 90%, meaning the delay tends to increase linearly when transmitting large files, such as images. QoS levels 1 and 0 had a delay of up to 155 ms (100 kB payload), and since the UAV could determine its path from just a few messages containing distance, angle, or another metric, this delay would be acceptable. QoS level 0 should be used in case of video transmissions, having a delay for all the cases of up to 135 ms on Wi-Fi.

For attack detection, DoS-type attacks were selected from the AWID2 dataset, and then the data were balanced to compare different ML algorithms based on decision trees with gradient boosting. Compared in terms of AUC, LightGBM was the best and even improved with the use of Bayesian optimization with 50 initial points and 500 iterations. XGBoost and CatBoost had similar results, with the problem of delay in training time. Tests were performed using both 16 and 4 columns of the dataset, where only 3 columns are float. With the reduction in the number of columns, there was a decrease in performance, but Set 1 of the columns still maintained a considerable result. Therefore, the two best models in the validation obtained a TPR of 0.97 for all columns and a TPR of 0.87 for Set 1, which was trained with the LightGBM with Bayesian optimization. This fact shows the achievement of high results even using a quartet of columns in training and testing.

Future work could be made to evaluate data transmission under GPRS, 4G, 5G, Sigfox, or other mobile protocols used on IoT. With the promising results obtained, the next step will be the implementation of this machine learning system into the platform itself, enabling attack detection and quick response that makes the platform safer to use. An appropriate strategy for the UAVs and platform behavior after detection of an attack will be implemented in the system, with tests and evaluations that will also be elaborated.

Field tests will be carried out to better evaluate the implemented platform and machine learning obtained. The tests will also allow the production of new data that can be useful and provide interesting feedback for making new decisions in the future. New tests will be implemented considering other algorithms that were not applied in this work; the use of unsupervised learning is a strategy that has been studied.

The availability of this platform for practical use is still being adopted, with the possible inclusion of other UAV models besides DJI Tello, such as Parrot Bebop 2 and DJI Spark, and the construction of a web application. The current status of the platform development can be followed at the URL provided in the data availability statement.

## Figures and Tables

**Table 1:** Features used and their descriptions and types.

Feature Name	Description	Type
frame.len	Frame length	Float
radiotap.channel.freq	Channel frequency value	Category
radiotap.channel.type.cck	Complementary code keying (CCK) flag	Category
radiotap.channel.type.ofdm	Orthogonal frequency division multiplexing (OFDM) flag	Category
radiotap.dbm_antsignal	Antenna DBM signal value	Float
wlan.fc.type	Type flag	Category
wlan.fc.subtype	Subtype flag	Category
wlan.fc.ds	Distribution system (DS) status flag	Category
wlan.fc.frag	More fragments flag	Category
wlan.fc.retry	Retry flag	Category
wlan.fc.pwrmgt	Power management flag	Category
wlan.fc.moredata	More data flag	Category
wlan.fc.protected	Protected frame flag	Category
wlan.duration	Duration value	Float

**Table 2:** Tasks measured on Ethernet and Wi-Fi connections.

	Ethernet		Wi-Fi	
**Action**	**Packets**	**Bytes**	**Packets**	**Bytes**
Connection to broker and topic subscription	23	7031	23	7020
Disconnection	4	253	4	252
Send telemetry payload (QoS = 0)	2	245	2	241
Send telemetry payload (QoS = 1)	3	328	3	329
Send telemetry payload (QoS = 2)	5	502	5	504
Send confirmation payload (QoS = 0)	2	225	2	221
Send confirmation payload (QoS = 1)	3	308	3	309
Send confirmation payload (QoS = 2)	5	482	5	484
Receive take-off command (QoS = 0)	2	232	2	232
Receive take-off command (QoS = 1)	3	327	3	323
Receive take-off command (QoS = 2)	6	561	6	554

**Table 3:** Correlation between payload and delay for each QoS level.

	Ethernet			Wi-Fi		
	QoS 0	QoS 1	QoS 2	QoS 0	QoS 1	QoS 2
*r*	0.952	0.968	0.931	0.951	0.936	0.925
$R^{2}$	0.906	0.937	0.866	0.904	0.923	0.855

**Table 4:** AUC of ML algorithms in training.

Algorithm	AUC
XGBoost	0.9981
CatBoost	0.9973
LightGBM	0.9982
LightGBM (with BO)	0.9983

**Table 5:** Performance of LightGBM (with Bayesian optimization) on each column set.

Set	F1 Score	AUC
1	0.97	0.9957
2	0.95	0.9916
3	0.95	0.9912
4	0.95	0.9913

## Data Availability

The UAV platform code is hosted in a github repository, which can be found at the URL https://github.com/silvamleandro/uav_platform (accessed on 24 August 2022). In this repository, there are also instructions on how to execute the controller and UAV codes, as well as the process for building the ML classifiers, all results, and extracted models.
